# Book Reviews

**Published:** 1803-05-01

**Authors:** 


### CRITICAL ANALYSIS
### OF THE
### RECENT PUBLICATIONS
### ON THE
### DIFFERENT BRANCHES OF PHYSIC, SURGERY, AND MEDICAL PHILOSOPHY.

*Beytrage zur Chemischen Kentniss der Mineral Korper;* i. e. Contributions to the Chemical Knowledge of Mineral Bodies; by MARTIN HENRY KLAPROTH, &c. third volume. Posen, 1802, 8vo. pp. 333.

IT is sufficiently known how much the analytical part of chemistry is indebted to the abilities and skill of the justly celebrated Klaproth, a name which will ever be respectfully pronounced in the annals of that science. The exertions of such men as Mr. Klaproth and Vauquelin, who, from the similarity of their merits, deserve to be nearly ranked, have much more contributed to the real advancement of the chemical art, than all the idle speculations of modern philosophers. The experience of centuries ought to have taught us, that the way which those gentlemen have traced out, is the only one by which it may arrive to that degree of perfection which it is capable of receiving through human abilities. Is it not by experiments and by facts, by the skilful management of the reagency, that modern chemists have so much succeeded in changing the face of this interesting science, as to render it useful to the arts, and conducive to the necessities and comforts of life? Why should we quit so sure a path towards perfection, and suffer ourselves to be led astray by the premature illusions of a licentious fancy? For such are the speculations of some German philosophers, who endeavour to extend this science beyond the region of facts and experience; whereby a prejudicial tendency in the study of Chemistry must necessarily be produced. The appearance of such works as the present, is therefore very consoling to an unprejudiced mind, and must be thankfully received by the public. As we suppose the two first volumes of this publication to be already in the hands of every chemist and mineralogist, we shall immediately turn our attention to the present, but lately published, the most interesting contents of which we beg leave to communicate to our readers. The preceding volumes contained about 72 chemical analyses, and these are continued in this volume to 116, some of which have already appeared in sundry periodical publications; the greater part, however, are quite new.

73. *Examination of the Transylvanian Gold Ores, Native Tellurium.* Under this name the author comprehends an ore, hitherto known by the appellation of *aurum paradoxum*, or *proble-maticum.* One thousand grains of it consist of 925,50 tellurium, 72,0 iron, and 2,50 gold. The chief characteristics of the metal of tellurium are the following: 1. Its colour resembles that of tin, approaching a little to the colour of lead. 2. It may be divided into lamellæ of a glossy surface, and on being suffered to cool gently it obtains a crystalline surface. 3. It is very brittle and harsh. 4. It has very little specific weight, namely, 6,113. 5. It is easily fusible, melting sooner than antimony, but later than lead. 6. It quickly takes fire before the blow pipe, burning with a light blue flame, which at its margin passes over to green. 7. It seems to enter into no combination with quicksilver. 8. Mixed and melted with sulphur, it forms a greyish, radiant ore. 9. It is perfectly soluble in nitric acid; the solution is quite clear, and not changed by the addition of water. 10. It makes likewise a clear solution with muriatic acid, particularly on adding a few drops of nitric acid. 11. On infusing one part of tellurium with 100 parts of concentrated sulphuric acid, an amethystine solution is formed, from which, however, the tellurium is separated by water in form of blackish flakes, and thus the beautiful colour disappears. 12. Sulphuric acid diluted with three parts of water dissolves the metal very easily, on adding a few drops of nitric acid: the solution is without colour and not decomposed by simple water. 13. Alkalis precipitate the oxyd of tellurium from all its solutions in acids with a white colour; but it is then very easily soluble in acids; and on adding more alkali than is absolutely necessary for making the precipitation, the oxyd is again dissolved, the alkali may have been caustic or not. The weight of the precipitated tellurium is increased by 20 per cent. if the alkali be caustic. 14. The solutions of tellurium in acids are not precipitated by prussiate of kali. 15. The sulphats of alkali precipitate the tellurium with a more or less brown colour. 16. Tincture of galls makes a yellowish precipitation. 17. Zinc and iron precipitate tellurium in metallic form, which is likewise the case with tin and antimony. Phosphorus even is covered with metallic lamella, if laid into a muriatic solution of tellurium. 18. Oxyd of tellurium is revived into metal on coal; but it is immediately volatilised and burned; the reduction of this metal must therefore be made in a closed vessel.— *Aurum graphicum*, 100 parts of which contain 60 metal of tellurium, 30 gold, 10 silver, &c.

75. *Chemical Analysis of the Scheele ore.* (Wolfram). The analyses of this metal is somewhat different from that of the late Mr. Scheele. The author found, in 100 parts, 77,75 yellow oxyd; 17,60 lime, 3 siliceous earth; and in scheelit, from Cornwall, 75,25 yellow oxyd, 18,70 lime, 1,30 siliceous earth, 1,23 oxyd of iron, and 0,75 oxyd of manganese.

76. *Chemical Analysis of the Gadolinit.* Having proposed the History of its Discovery and Chemical Analysis, the author proceeds to give a description of this mineral, and adds his own analysis, according to which 100 parts of the gadolinit contained 59,75 ytter earth, 21,25 siliceous earth, 17,50 black oxyd of iron, 0,50 aluminous earth, and 0,50 water. For separating the iron the author made use of the succinat of natron. The experiments of Mr. Klaproth manifestly show the identity of the ytter earth, and its real difference from the glycine.

77. *Chemical Examination of the Egyptian Natron.* Besides the earthy admixtures, this alkali contains 163 carbonat of natron, 104 dry sulphat of natron, 75 dry muriat of natron, and 158 water.

78. *Chemical Examination of the Radiant Natron.* The natron found in the natron lakes of Egypt, frequently occurs in crystaline masses of a considerable hardness, and it never falls into a powder, on account of which property Mr. K. thought it worthy of being examined. He found in 100 parts of radiant natron of Tripolis, 22,50 water of crystallisation, 38 carbonic, 37 pure natron, and 2,50 sulphat of natron. The great proportion of carbonic acid seems therefore to account for that property of the Egyptian natron.

79. *Chemical Analysis of native Sal Ammonia.*

80. *Chemical Examination of the Sassolin.* The native sal sedativum, which bears the name of sassolin, consists of a white brittle salt with yellow spots, the principal constituent of which is free boracic acid. Mr. Mascagni found it at the margin of a hot spring, near Sasso in Siena. It consists of 86 boracic acid, 11 sulphat of borax, and 3 sulphat of lime, with some iron particles, &c.

82. *Chemical Analysis of the Elastic Bitumen.* This substance is from the lead mine, Odin, near Castleton, in Derbyshire. The products obtained by its analysis were 38 cubic inches of carbonated hydrogen gas, 4 cubic inches carbonic gas, 73 grains of bituminous oil, 1,50 acidulous water, 6,25 coal, 2 lime, 1,50 siliceous earth, 0,75 oxyd of iron, 0,50 sulphat of lime, 0,25 aluminous earth. This analysis, however, gives no explanation of the singular properties of this bitumen.

84. *Chemical Analysis of the Honeystone.* This excellent analysis proves that the honeystone consists of a peculiar acid, and 106 parts of it contain 46 melilithic acid, 16 aluminous earth, and 38 water of crystallisation.

85. *Chemical Analysis of the Umbra.* The true umbra of the island of Cyprus, consists of 48 oxyd of iron, 20 oxyd of manganese, 13 siliceous earth, 5 aluminous earth, 49 water.

86. *Chemical Examination of a muriated Lead Ore, or Lead Spar.* The existence of such an ore was still uncertain, but is proved by the present analysis. The author received this mineral from Derbyshire, in which he found 85,50 oxyd of lead, 8,50 muriatic muriatic acid, and 6 carbonic acid, with a little water of crystallisation. He made use for this analysis of the purest kali, which he prepared in the following manner: Having dissolved pure nitre in distilled water, he added nitriate of silver, and having filtrated the liquor, evaporated it to dryness, and burned it in a clean iron crucible with pure coal; and being dissolved and neutralised with nitric and acetic acid, it was not in the least rendered turbid by the solutions of silver; whence it appears that the muriatic acid, discovered in that lead ore, did not originate in the kali, which was used for the analysis.

87. *Chemical Analysis of Phosphated Ores.* Amongst the different lead spars which are here examined, we observe that the yellow lead ore from Wanlock-Head, contains 80 oxyd of lead, 18 phosphoric acid, and 1,62 muriatic acid.

88. *Chemical Analysis of Sulphated Lead Ores.* 1. *From Anglesea,* 71 oxyd of lead, 24,80 sulphuric acid, 2 water of crystallisation, 1 oxyd of iron.—2. *From Lead Hills,* 70,50 oxyd of lead, 25,75 sulphuric acid, 2,25 water of crystallisation.

89. *Chemical Examination of the Lamellar, White Lead Ore from Lead Hills.* About 77 oxygen.

90, &c. *Analysis of several Antimony Ores from the Harz.*

94. *Chemical Analysis of the Olive Ore.* 50,62 oxyd of copper, 45 arsenic acid, and 3,50 water of crystallisation.

95. *Chemical Analysis of the Muriated Copper.* 73 oxyd of copper, 10,1 muriatic acid, and 16,09 water of crystallisation.

96. *Chemical Analysis of the phosphated Copper.* 68,13 oxyd of copper, 30.95 phosphoric acid.

97. *Chemical Examination of the Kryolithos.* 36 natrum, 24 aluminous earth, 40, fluoric acid.

98. *Chemical Analysis of the Beryll,* agrees almost with that given by Mr. Vauquelin. The author found in 100 parts of this fossil 66,45 siliceous earth, 16,75 aluminous earth, 15,50 glycine, or beryll earth, and 0,60 oxyd of iron.

99. *Chemical Analysis of the Emerald.* 100 parts contained 68,50 siliceous earth, 16,75 aluminous earth, 12,50 glycine, 0,25 lime, 1 oxyd of iron, 0,30 oxyd of chromium.

100. *Chemical Analysis of the Klingstone,* which is the hardest kind of fieldspar.

101. *Chemical Analysis of the Basaltes,* 100 parts of which contain 44,50 siliceous earth, 16,75 aluminous earth, 20 oxyd of iron, 9,50 lime, 2,25 magnesia, 0,12 oxyd of manganese, 2,60 natrum, 2 water.

102. *Chemical Analysis of the Pitchstone.* One hundred parts consisted of 78 siliceous earth, 14.50 aluminous earth, 1 lime, 1 oxyd of iron, 0,10 oxyd of manganese, 1,75 natrum, and 8,50 water.

103. *Addition to the Chemical Analysis of the Pitchstone.* It contains natrum and kali at the same time, which is very remarkable.

104. *Chemical Analysis of the Zirkon, from Norway.* 65 zirkon earth, 33 siliceous earth, 1 oxyd of iron.

105. *Analysis of the Madreporit.* 100 parts contained 93 carbonat of lime, 0,50 carbonat of magnesia, 1,25 carbonat of iron, 0,50 coal, 4,50 siliceous sand, and some traces of oxyd of manganese.

106. *Chemical Examination of the Pharmacolith.* 50,54 arsenic acid, 25 lime, and 24,46 water.

107. *Chemical Examination of the Scorza.* This name is given in Wallachia and Transsylvania to a sandy mineral of a yellow greenish colour, in small roundish grains, 100 parts of which contained 43 siliceous earth, 21 aluminous earth, 14 lime, 16,50 oxyd of iron, 0,25 oxyd of manganese.

108. *Chemical Analysis of the fibrous Sulphat of Barytes.*

109. *Chemical Examination of the Tabular Spar,* (Tafelspath.) 50 siliceous earth, 45 lime, and 5 water.

110. *Chemical Analysis of the Miemit.* This fossil bears its name from its native place, Miemo, in the duchy of Toscana, whers it was first discovered in the year 1791, by Mr. Thomson. One hundred parts of it contain 53 carbonat of lime, 42,20 carbonat of magnesia, 3 carbonat of iron, and some traces of manganese.

112. *Chemical Analysis of the fibrous Grey Ore of Manganese.* 1. From the Harz. 100 parts contained 90,50 black oxyd of manganese, combined with the maximum of oxygen, which it is capable of retaining in fire. 2 From Moravia, 89 black oxyd of manganese, 0,50 water, 10,25 oxygen.

114. *Chemical Examination of the Terreous Black Ore of Manganese.* 68 brown oxyd of manganese, 6,50 oxyd of iron, 1 coal, 1 barytes, 8 siliceous earth, 17,50 water.

114. *Chemical Examination of the Asphaltes from Albania.*

115. *Chemical Analysis of the earthy Bovey Coal.*

116. *Chemical Analysis of the Hungarian Pearl Stone.* One hundred parts of this fossil contain 75,25 siliceous earth, 12 aluminous earth, 1,60 oxyd of iron, 0,50 lime, 4,50 kali, and 4,50 water.

Such are the contents of this interesting work, the continuation of which will undoubtedly be very much desired by Chemists as well as Mineralogists.

---

*A Popular View of the Structure and Economy of the Human Body, &c. by* John Feltham. London, 1803, pp. 452. 8vo.

Though the work before us will hardly excite the attention of the greater part of *our* readers; yet as the author professes to have performed a task which has long been considered by some as a desideratum in the study of anatomy, we shall give a short view of its leading contents.

The object of this work is to give such a compendious view of the structure and economy of the human body as to be within the reach reach and comprehension of every person of tolerably liberal education; unrestricted to age, sex, or profession. The present times are not very unfavourable to such a plan; an excellent institution, patronized by rank and fashion, and supported solely by private contributions, already bears an honourable testimony to the zeal for the advancement of science existing in this metropolis; and it is no very unreasonable expectation to entertain, that the study of Anatomy, properly directed, may in time become a part of the regular education of youth, whom it cannot fail to interest, delight, and improve. The author does not hesitate to recommend this study to women, both "to enable them to take proper care of their own health, and to make them rational superintendants of their infants, parents, and husbands. Till of late years," he observes, "women were kept in a state of Turkish ignorance as to the objects and means of intellectual improvement; every source and channel of acquiring knowledge was obstructed, if not discountenanced, by fashion: Books of science were replete with a jargon of unintelligible terms, by which ignorance was often mysteriously and pompously veiled and shielded from public contempt; but now, by a happy revolution in the public taste and judgment, writers offer these discoveries to the public in distinct terms, which every body may understand; technical language is no longer allowed to supply the place of real knowledge, and the art of communicating instruction has been carried to the greatest perfection."

Another and a very laudable object which the author proposes, is to exhibit such a view of the anatomy of the human frame, "as may be calculated to inspire veneration and love for the Supreme Artificer;" and certainly, of all sciences, Anatomy is the richest in those examples and illustrations of supreme contrivance, which immediately appeal to the understanding, and interest the imagination. Considered in this point of view, however, it is not a very easy task to introduce with grace and elegance these appeals to the religious sense derived from the inspection of the works of Nature. When not intended to form the basis of logical induction, (as in the late admired work of the learned and philosophic Paley) they should flow easily and directly from the subject, not constantly recurring nor ostentatiously prominent; and the rich but turgid style of the celebrated author of the Meditations, can be but ill engrafted on that of simple and perspicuous narrative, in which instruction should be conveyed.

The introductory chapter of the present work contains, after the usual compliment to modern improvements in science, (among which the *metallic tractors* are represented on equal terms with Galvanism) a slight and trifling sketch of the history of Anatomy, to which succeeds a clear and neat explanation of the parts of the human body, together with their general uses.

In the second chapter a familiar view of the structure of the human body is given. Though by no means compleat, it is elegant and interesting; the important processes of digestion and nutrition

 are are first explained, and the chapter concludes with an animated description of the senses, principally taken from Hervey.

Osteology is a part of Anatomy well calculated to exercise the powers of description of any writer who wishes to make Anatomy entertaining as well as interesting. By constantly recurring to the uses of parts, and the admirable contrivances for giving support, protection, and mobility, osteology may be rendered in no small degree attractive, especially to those who possess some knowledge in mechanics. This is the subject of the third part of the present work. The description is clear and intelligible, but is far from exhibiting such a view of osteology as the limits of the chapter would well allow of; and this unnecessary brevity much diminishes the force of the numerous appeals to the devotional sense, by rendering them too general, and not sufficiently appropriate.

The fourth chapter contains a general description of the structure of the muscles, and of the supposed cause of muscular motion, which latter is thus described, "If a muscle is pricked or irritated it immediately contracts. This is called its irritable principle, and this irritability is to be considered as the characteristic of muscular fibres, and may serve to prove their existence in parts that are too minute to be examined by the eye. This power, which disposes the muscles to contract when stimulated, independant of the will, is supposed to be inherent in them, and is therefore named *vis insita;* this property is not to be confounded with elasticity, which the membranes and other parts of the body possess in a greater or less degree in common with muscles; nor with *sensibility*, for the heart, though the most irritable, seems to be the least sensible of any of the muscular parts of the body. After a muscular fibre has contracted, it soon returns to a state of relaxation, till it is excited afresh, and then it contracts and relaxes again; such a contraction may likewise be produced by irritating the nerve leading to a muscle, although the nerve itself is not affected."

An account of the nervous system follows, in the fifth chapter, which is accurate and interesting in the merely descriptive part, but short and unsatisfactory in the theoretical opinions concerning nervous influence.

The sixth chapter, which contains a description of the blood-vessels, heart, and circulating system, is perhaps the most complete in the volume, and appears to us fully to answer to the plan of the author, in exhibiting, in a moderate compass, a clear view of the subject, unincumbered with many technical terms, and readily acquired by the student. The description of the heart will serve as a good example of the author's manner of treating the subject.

"As the heart is the fountain from which this vital fluid issues, it may be necessary to say, that the heart is a hollow, involuntary muscle, of a conical shape. Its basis, from which the great vessels arise, is covered with fat, and it has two hollow and fleshy appendages called Auricles. The heart includes two cavities, divided from each other by a fleshy septum; one of these is called the right, right, the other the left ventricle, though perhaps they might more properly be termed the anterior and posterior ventricles. From the contraction of one of these ventricles the blood is forced into vessels, which carry it into the remotest part of the system; these vessels are called arteries; and it is brought back again by another set of vessels called Veins, to the heart, so that the same force which sends it forward, also beings it back again. The right ventricle is not quite so long, though somewhat larger than the left, but the latter has more substance than the former; and this seems to be, because it is intended to transmit the blood to the most distant parts of the body, whereas the right ventricle only distributes it to the lungs. The heart is usually described as of a pyramidal form, but one of its sides is rather flat, and this is placed downward; the right side is placed in front: but in brutes, the right ventricle is on the right side, and the left on the left, from whence their names.

"The auricles are situated at the basis of the heart; these muscular bags, corresponding with the ventricles, are like them divided into right and left; from their externally appearing like ears, they derive their name of auricles. The heart, in contracting, throws the blood from its ventricles into the pulmonary artery, and the great artery termed *aorta*, it then relaxes and receives a fresh supply, from two large veins, which are the pulmonary veins, and the vena cava; the principal distribution of these are as follows."

In the two following chapters the author describes, first, the *Internal Parts of the Body*, including under this title, the contents of the three great cavities of the cranium, thorax, and abdomen; and, secondly, the *External Divisions of the Body*, containing the anatomy and functions of the skin, external organs of respiration, and of the senses.

The subject of the ninth chapter is of all others the most difficult to be well and properly treated of in a popular work like the present; it is, *The Structure of the Human Form, Male and Female.* The distinction of sex, and the very important branch of anatomy connected with it, must claim a large share of attention in the study of the animal frame; but the author, well aware of the difficulty of the subject under his plan of general information, has (probably with great propriety) chosen rather to leave his work imperfect, than to introduce any thing which could be thought objectionable by the most delicate parent or instructor. The present chapter, therefore, only treats of the differences of form and features, and of general character, both in external organization, and in moral habits, tastes, and inclinations, which exist in the different sexes. These are described with elegance and delicacy.

The tenth and eleventh chapters, which conclude the volume, contain a slight view of comparative anatomy, which however will be perused with interest and entertainment.

The author has, we think, succeeded in making an useful and entertaining compendium of anatomy; but we must add, that with the anatomical knowledge which he appears to possess, and the powers for accurate description, and for clear and forcible condensation of an extensive subject, which sometimes appear in these pages, a greater attention to order and arrangement, and fewer digressions, would have rendered the work much more valuable, instructive and entertaining. It must give disappointment, even to the most pious reader, when in search of matter of fact and actual information, to be constantly diverted from his enquiry by devotional reflections; which, however excellent in themselves, lose much of their force by anticipating those subjects which they would have followed with peculiar propriety. The author does not reject the assistance of the Muses to decorate his labours, and the poetical description which he occasionally introduces is generally highly elegant and well selected. As our Retrospect is not often enlivened by such ornaments, we shall quote for our reader's entertainment the following original poetical address.

### TO THE HUMAN COUNTENANCE.

MYSTIC source of strange expression,
Fairest link of nature's chain,
Stamp'd with God's divine impression
O'er his mighty works to reign;

Whence, O say, thy mighty treasure?
From what wide, unknown abyss,
Ever yield'st thou endless pleasure,
Speechless, gentlest, wildest bliss?

Is it in thy front aspiring,
Where the virgin lily blows,
While, with living purple firing,
Spreads the gentle, blushing rose?

Or, with pensive lustre streaming,
Where yon sparkling glories rise,
Sweetly sad, like Cynthia beaming,
In thy love-inspiring eyes?

Is it in yon bed of roses,
Breathing thousand odours round;
Lucy's lip, where love reposes,
In etherial fetters bound?

Or, in yonder winding dimples,
Magic cells of fairy art,
Where the elfin, culling simples,
Brews his spell upon the heart?

"Cease, O cease thee, sightless creature,"
Thus I hear thee stern reply,
"'Tis not in *one* wizard feature
"My enchanting sources lie;

"Neither

"Neither yet, where gently flowing,
"Each in each congenial run,
"Softly blending, fading, glowing,
"Sweetly struggling into one.

"But in that mysterious union,
"Secret source of strange control,
"In that sweet, divine communion
"Of the features and the soul.

"Ponder then, O child of pleasure;
"Haste to seize on *Virtue's* grace,
"Woud'st thou have the magic treasure
"Of a love-inspiring face."

---

*Observations on a late Publication of Dr. Pearson, entitled, An Examination of the Report of the Committee of the House of Commons, on the Claims of Remuneration for the Vaccine Pock Inoculation. With an Appendix, containing some Reflections on an Article in the Critical Review for October last, respecting the same Publication.* By HENRY HICKS. London, 8vo. pp. 74.

WHEN we mentioned Mr. Creaser's pamphlet, (p. 286 of this vol.) on the same subject as the present, we passed it over very shortly, believing that the public opinion was already decided on this point. The appearance, however, of Mr. Hicks's work, and some other recent publications, convince us that there are some who think otherwise, though the number may be small. The object of both these pamphlets is the same, viz. to vindicate the conduct and fame of Dr. Jenner, and to answer Dr. Pearson: but the style and manner in which this is done, are very different. Mr. Creaser treats the subject with the calmness of philosophical investigation, and seems only desirous of establishing the truth. Mr. Hicks does not content himself with merely vindicating his friend, but frequently attacks his opponent with considerable severity. This he justifies on the ground of the long and intimate friendship which has subsisted between Dr. Jenner and the writer. "I feel," says he, "a considerable degree of reluctance in addressing the public upon a subject which has so much occupied its attention; and I certainly should not have undertaken such a task, if the reputation of a man, with whom I am proud and happy to say I have ever lived in the closest and most uninterrupted friendship, had not been attacked in the most shameless and unjustifiable manner by Dr. Pearson, in a late publication, entitled, 'An Examination of the Report of the Committee of the House of Commons, on the Claims of Remuneration for the Vaccine Pock Inoculation.'

"Before I took up my pen, I wrote to my friend, Dr. Jenner, to ask him, whether he intended to make any Reply to Dr. Pearson's invidious attack upon his reputation. His answer was, as I expected expected it would be) that as he had never yet thought it worth his while to notice any of his former remarks, he should now pursue the same line of conduct, and treat his present publication with silent contempt.

" Under the impression of this determination, with feelings of the most affectionate regard for Dr. Jenner, I cannot think it necessary to make any further apology for having thus come forwards in his defence."

The only part of the evidence brought forward by Dr. P. which could apply to Dr. Jenner, we think was that furnished by the Rev. Mr. Drewe; and this, if it had gone as far as Dr. P. seemed to hope and expect, could only have affected his claim to priority of idea, not to rénumeration; unless it had been proved that Dr. Jenner received his information from the Rev. Mr. Drewe, which has never been insinuated. Mr. Hicks thus states the amount of that evidence.

" In page 4, of his first publication, Dr. Pearson gives Dr. Jenner the greatest merit 'for the industry of his researches, in ascertaining the invaluable fact of Cow-pox being an effectual preventive of Small-pox;' and he tells us, that the late Mr. John Hunter communicated to him the information he had received from Dr. Jenner, upon this subject, about nine years ago. Dr. Pearson, himself, in his lectures, always mentioned the circumstance as reflecting credit upon Dr. Jenner, and as a striking proof of his ingenuity; but when this declaration was made, the spirit of enmity had not taken possession of his mind.

" Page 6, Dr. Pearson says, " that on conversing with Sir George Baker, Bart. concerning the Cow-pox rendering people unsusceptible of the variolous disease, Sir George observed, he had been informed of the same fact, in some papers on the Cow-pox, communicated to him many years ago; but as the statement did not then obtain credit, it was not published. After a fruitless search for these papers, Sir George, whose zeal for the improvement of physic did not forsake him on this occasion, authorized me to write to his relative, the Rev. Herman Drewe, of Abbots."

Dr. P. next gives us Mr. Drewe's letter, dated July 5, 1798, by which we are informed, that a Mr. Bragge, who inoculated his parish for the Small-pox, charged with a superabundance of matter three women that had had the Cow-pox, who completely resisted the infection; and who were not in the least disordered, though they constantly associated with other patients who had been inoculated for the Small-pox with effect. He also adds, that there were thirteen similar instances in the same neighbourhood. Mr. Drewe then tells Dr. Pearson from whom he received all his information, and concludes by saying, 'that he had not thought of the matter since, and that as his letter on the subject had escaped Sir George Baker's search, so had many particulars escaped his recollection.' Was ever any thing more vague or unsatisfactory than all this? Can it be supposed that Sir George Baker, a man of of such active scientific enquiry, would have destroyed or mislaid papers,* which tended to throw so much new light on so important a branch of physiology?

"I wish my readers to compare this statement with that which Dr. Pearson adduced before the Committee, and which he has detailed from page 17 to 29.

"I would ask Dr. Pearson, why this correspondence, which is so slightly mentioned in his first publication, swells into so much consequence upon the presentation of Dr. Jenner's petition, and why he anxiously labours to convert it into the means of depriving him of the merit of being the Discoverer of Vaccine Inoculation? If truth is to dictate the reply, this question may soon be answered.—We can only refer it to that spirit of hostility against Dr. Jenner's reputation, which so strongly marks all his proceedings.

"We next remark a quotation from Mr. Drewe's letter, which stands in direct opposition to what Dr. Pearson endeavours to support, from a correspondence upon which I have before made some observations. Mr. Drewe says, 'Mr. Bragge and I endeavoured to try the experiment of Inoculation with the matter of Cow-pox, but from the scarceness of the disease, and the unwillingness of patients, we were disappointed.' Where then are all the boasted experiments of these gentlemen? What I have already said is sufficient to shew the real character of Dr. Pearson; and I trust I shall be able to prove, in my subsequent remarks, notwithstanding the panegyric which Dr. Pearson has lavished upon himself, that he has by no means improved the practice; that he has not established a single new fact; and, that he has totally failed in his invidious efforts to blast the well-earned fame of Dr. Jenner.

"I shall now proceed with my observations upon Dr. Pearson's examination of the Report of the Committee.

"The very first page presents something so insidious, that it ought not to pass unnoticed: Dr. Pearson says, 'that he can with truth declare, Dr. Jenner to be the *first discoverer to the public* of Vaccine Inoculation, agreeable to the acknowledgement contained in his first publication.' He, then, thinks it necessary to explain what he means by *discoverer to the Public;* in which he betrays a most determnied opposition to Dr. Jenner's claim to originality, as well as to the grounds upon which the Committee thought he had founded his title to public remuneration. The first emotion which is excited in an ingenuous mind, by this hypocritical explanation, must be a strong feeling of contempt, but this will be immediately succeeded by the stronger impulses of indignation.

Though Dr. Pearson labours and toils throughout his whole book

---

* I have heard that Sir George Baker declared he burnt the papers designedly. A pretty convincing proof of what value they were in his estimation.

book to detract from the propriety of all Dr. Jenner's claims, any unprejudiced person will at once perceive, how totally he has failed in his design, and how much he has exposed his own want of *candor* and *justice*.

"I cannot help frequently recurring to a comparison of some of Dr. Pearson's own statements; and, perhaps, this is the shortest way of exposing their fallacy. In page 2, of his late work, he tells us, 'that no fact has informed him that human society would have been at this hour in the possession of the blessings of Vaccine Inoculation, had it not been for Dr. Jenner's publication of the treatise on the causes and effects of the Variolæ Vaccinæ, in June, 1798.' "Does not this confession comprize every thing that can be advanced upon the subject? and, after such a confession, are not all Dr. Pearson's arguments against Dr. Jenner's claim to originality perfectly nugatory? It seems almost to supersede the necessity of going further into the question. Does this at all agree with Dr. Pearson's remarks on the correspondence of Mr. Drewe, &c.? Were not these correspondences brought forwards with the view of detracting from Dr. Jenner's merits? There is something so invidious (I had almost said malevolent) in the whole of this transaction, that I know not how to employ language sufficiently strong to express my reprobation of it. If all these facts had existed, as related in these letters and in Mr. Nash's evidence, how are they to be reconciled to Dr. Pearson's declaration which I have just before recited?"

---

*On the Inutility and Dangers of Vaccine Inoculation, proved by Facts, by* Cit. GOETZ, *of Paris.* French 8vo. pp. 90. Paris, 1802.

THE following passage, which we have translated as literally as possible, will shew the merit of this extraordinary production as well as the author's knowledge of the subject upon which he has written.

"The vaccine virus must act in one or other of these two ways; either it must *destroy* the germe of the small-pox, which lies latent in the constitution of every one who has not had the disease; or it must *neutralize* this germe."

The former supposition he thinks improbable, and on the latter he says, "Will you pretend to neutralize to the germe of the small-pox by inserting vaccine virus alone? No, doubtless; since it is proved that the matter you employ, and to which you have been pleased to give the name of vaccine virus, is nothing else but a *mixture of this last with the matter of small-pox.* What is the result of this mixture, &c.

---

*Facts and Observations respecting the Air-Pump Vapour-Bath, in Gout, Rheumatism, Palsy, and other Diseases.* By R. BLEGBOROUGH, M.D. 12mo. pp. 150. London, 1803.

THE utility of applying heat, moisture, and a varied pressure of the atmosphere, to the surface of the body, in the manner here recommended, mended, is shewn in a variety of cases of gout, rheumatism, palsy, cutaneous affections, and chronic pains. The whole is interspersed with observations and inferences calculated to extend its use to many other complaints in which it has not yet been sufficiently tried: such as tetanus, amenorrhœa, dropsy, chilblains, &c. The work concludes with an appendix, explaining the principles on which the apparatus acts on the human body, and the means by which the invention was brought to its present degree of perfection.

---

*The Domestic Medical Guide, or Complete Companion to the Family Medicine Chest; comprising a copious Account of Diseases, and the most rational Mode of Treatment, the Management of Children, Treatment of Poisons, of drowned Persons, and the Method of destroying Contagion.* By R. Reece. 8vo. pp. 302. London, 1803.

This work is divided into two parts. The first contains an account of such medicines as families residing in the country should be provided with; more especially if at a distance from medical aid. The utility of such a provision, we believe, is generally acknowledged; and the difficulty of preventing an improper use of it, as generally confessed. Mr. R. endeavours to prevent family quackery by suitable cautions and directions; and if these are attended to, we think his medicine chest may be made a very useful addition to the furniture of a country mansion. There can be little doubt that many valuable lives are lost for want of timely assistance; and where it is often difficult to get a practitioner in less than eight or twelve hours, this must frequently happen. In cases of poisons swallowed, of drowning, burns, and fits, as well as acute diseases, the aid must be speedy to be effectual. Under this impression, we have no hesitation in recommending Mr. R's collection of family medicines, and his directions for using them when necessary.

The Second Part contains, an account of poisons, suspended animation, nursing, and a description of the diseases which occur in this country, with their distinguishing characters and treatment.

The observations on quack medicines are well calculated to guard the public from their pernicious effects.

